# Effectiveness of Different Categories of Light Oils in Partially Reactive Crumb Rubber-Modified Asphalt

**DOI:** 10.3390/ma18081871

**Published:** 2025-04-19

**Authors:** Dean Wen, Dongdong Ge, Yantao Wang, Songtao Lv, Qian Liu, Shuxian Liu

**Affiliations:** 1 CCCC-SHB Fourth Engineering Co., Ltd., Luoyang 471013, China; 18848968087@163.com (D.W.); 15672891123@163.com (Y.W.); 2 National Key Laboratory of Green and Long-Life Road Engineering in Extreme Environment, Changsha University of Science & Technology, Changsha 410114, China; lst@csust.edu.cn (S.L.); lq@stu.csust.edu.cn (Q.L.); lsx@stu.csust.edu.cn (S.L.); 3 National Engineering Laboratory of Highway Maintenance Technology, Changsha University of Science & Technology, Changsha 410114, China

**Keywords:** asphalt, crumb rubber, light oil, rheological property, microscopic analysis

## Abstract

Rubber-modified asphalt (RMA) faces several challenges, including poor workability, difficult construction, and high energy consumption. The incorporation of renewable light oils offers a promising solution to address issues such as high viscosity and elevated carbon emissions in asphalt modified with a high dosage of rubber powder. The investigation of light oil and rubber powder composite-modified asphalt under low-temperature (160 °C) and short-term (30 min) shear processes is essential for understanding its rheological behavior and modification mechanism. This study explores composite-modified asphalt prepared with four types of light oils (fatty acids, aromatic oil, tall oil, and paraffin oil) at dosages of 10% and 15%, combined with 20% rubber powder. Conventional penetration and viscosity tests were carried out to assess the overall physical properties of the composite-modified asphalts, while rheological tests were conducted to examine their performance at high temperatures. Fourier transform infrared spectroscopy (FTIR) and fluorescence microscopy (FM) were employed to explore the interaction mechanisms that occurred between the light oils, rubber powder, and asphalt. The results suggest that the addition of various light oils leads to a reduction in the viscosity of rubber-modified asphalt, with the extent of reduction varying across different oils. Notably, 10% tall oil demonstrates the most significant reduction in viscosity while also facilitating the dissolution of rubber powder. The high-temperature PG-grade rubberized asphalt improved with the incorporation of light oils, with 5% tall oil yielding the highest PG grade of PG 82-34. FTIR analysis confirmed that light oils and rubber were physically blended in the asphalt, with the light components of the oils being absorbed by the asphalt. FM observations revealed that light oils promote the swelling of rubber particles, with the rubber particles fully swelling in tall oil. Considering the reduction in viscosity, the performance at both high and low temperatures, elasticity, and the extent of rubber particle swelling, tall oil is identified as the most effective material for preparing light oil–rubber composite-modified asphalt using the low-temperature, short-term shear process.

## 1. Introduction

Asphalt pavements, with their exceptional performance, have become an indispensable component of modern transportation infrastructure. Driven by the concept of sustainable development, asphalt products are advancing towards more environmentally friendly and sustainable solutions. Waste rubber, a material often referred to as “black pollution” due to its environmental impact, has been refined into rubber powder and applied to asphalt modification. This not only imparts new properties to asphalt but also provides an important avenue for the harmless disposal of used tires [1]. However, rubber powder-modified asphalt (RPMA) faces several challenges, including high viscosity, difficulties in construction, and high energy consumption. These issues have prompted researchers to explore renewable resources to optimize its performance. Lightweight oil, a renewable material with a wide range of sources, can effectively address the shortcomings of rubber powder-modified asphalt, making it a promising new modifier in the formulation of RPMA [2]. This study examined the impact of various types and dosages of lightweight oil on the characteristics of rubber–lightweight oil composite asphalt.

As a road construction material that combines environmental considerations with better road performance, RPMA demonstrates significant development potential and broad application prospects [3].

When rubber crumbs are blended into asphalt, they cause expansion, and their effective components form a network structure through crosslinking reactions, resulting in a significant enhancement of the thermal properties as well as flexibility in cold-temperature conditions [4]. The size of the rubber powder particles influences their solubility in asphalt; finer particles tend to swell more in asphalt, while ultra-fine rubber powder raises the viscosity of asphalt, thereby complicating the construction process [5]. The preparation of RPMA primarily utilizes the high-shear mixing method. The recommended rubber particle content is 10–20%, with a shearing time of 60 min, shearing temperature of 180–200 °C, and shearing speed of 1500–5000 r/min [6]. The rubber powder content is positively correlated with the storage stability of the modified asphalt [7].

However, conventional asphalt modified with vulcanized rubber powder exhibits several issues, including inadequate high-temperature storage stability, susceptibility to segregation, elevated viscosity, and challenges during construction. Desulfurization technology can be employed to obtain highly active rubber powder, thus reducing the viscosity of asphalt modified with rubber [8]. Zhang et al. [7] conducted Fourier transform infrared spectroscopy (FTIR) experiments on rubber-modified asphalt after desulfurization using an OD desulfurization agent. The results indicated that modifying asphalt with rubber powder primarily involves a physical alteration, without the disruption of existing bonds or the formation of new chemical connections. Jiao et al. investigated the mechanisms of desulfurization and breakdown of rubber powder through a series of macroscopic and microscopic tests [9]. The findings indicated that, as the degradation level of the rubber particles increased, the lighter components of the rubber became more soluble in bituminous material, elevating the dispersion of the bitumen.

High-content rubber powder-modified bitumen and its mixtures offer excellent service performance. However, an excessively high rubber powder content increases viscosity, reduces workability, and compromises thermal storage stability, ultimately diminishing the water-resistant stability. Ma et al. demonstrated that after adding stabilized rubber granules, the temperature sensitivity response was enhanced. With a rubber content of 30%, the flowability was dramatically reduced at 135 °C [10]. Mohamed et al. observed the effectiveness of rubber particles and recycled bitumen through a series of experiments, which showed a notable improvement in bonding strength, rutting resistance, creep resistance, and colloidal homogeneity [11].

In summary, while RPMA offers good elevated-temperature capabilities and performance under cold conditions, its high viscosity, poor processability, and other issues seriously limit the development of asphalt formulations with a significant rubber composition. Therefore, reducing the viscosity and enhancing the flow behavior of RPMA is crucial for construction.

Lightweight oil generally refers to hydrocarbons; in the petroleum refining industry, it can refer to light distillates or light oil products [12]. Lightweight oil offers several notable advantages, such as enormous reserves, environmental sustainability, wide availability, and a relatively low cost [13]. According to research from the National Center for Asphalt Technology (NCAT), the primary types of lightweight oil used in modified asphalt include paraffin oil (refined waste lubricating oil), aromatic oils (refined crude oil containing polar aromatic components), fatty acids (hydrocarbon engineering products used for asphalt modification), and tall oil (a byproduct of the paper industry) [14].

Samieadel et al. investigated how different concentrations of paraffin oil influence the characteristics of asphalt binders at the nanoscale. The findings showed that as the concentration of paraffin oil increased, the average size of the bitumen binder structure decreased and the number of bitumen clusters increased [15]. Liu et al. explored the impact of exhausted engine oil (WEO) on bitumen performance, finding that WEO addition reduced the percentage of large molecules, significantly decreased asphalt viscosity and application temperature, and had a negative effect on rutting resistance, while improving fatigue performance [16]. Yesilcicek et al. chemically synthesized a novel asphalt additive by combining paper-industry byproduct tall oil with boric oxide. Macrolevel asphalt tests showed that the modified asphalt exhibited improved workability and reduced temperature sensitivity. Additionally, the rutting resistance and fatigue performance were enhanced, although at higher dosages the low-temperature performance was severely impacted [17]. Seidel et al. mixed a fatty acid extracted from soybeans with asphalt. Rheological tests revealed that the soybean-acidified soap stock not only softened the asphalt binder, reducing rigidity, but also improved its high-temperature performance [18]. Haghshenas et al. investigated the relationship between the gel index and chemical rheology in asphalt modified by five types of rejuvenators, including paraffin oil, aromatic extracts, naphthenic oil, fatty acids, and tall oil. The study showed a strong linear correlation between the rheological behavior under chemical action of the binder and the gel index and that rejuvenators could alter the softening and long-term properties of the modified binders based on their chemical composition. While all recycled agents softened the binders, only a few could restore the rheological properties of the binders [19]. Ye, Q. et al. prepared composite rejuvenators (CD) using cashew phenol and distilled tall oil. Macro- and microscopic tests confirmed the efficacy of CD, which outperformed single-component rejuvenators (soybean oil and aromatic oil) in terms of restoring asphalt properties [20].

Numerous researchers have demonstrated that mixing lightweight oil with bitumen is able to compensate for the volatile components present in the asphalt, thereby improving viscosity and workability [21,22,23]. The impact of lightweight oil from different sources on the elevated and reduced temperature extremes of asphalt varies significantly. The capabilities of single lightweight oil-altered asphalt are often insufficient to meet the demands of modern road construction. Thus, it is necessary to propose targeted modification methods to address the shortcomings of existing lightweight oil-modified asphalts.

Lightweight oil can promote suitability and processability, besides the long-term steadiness of rubberized bitumen during the construction process [24]. A large number of studies signaled that incorporating light oil within rubber-altered asphalt significantly improved the cool-temperature properties of the asphalt and reduced the overall viscous characteristics, thereby facilitating construction [2,25,26,27]. The integrated influence of lightweight oil and rubber greatly augmented the anti-aging properties of the asphalt, with the improvement in UV aging resistance being more significant than that in thermal durability [28,29,30,31].

Xu et al. carried out a series of performance evaluations on high-dissolution rubber asphalt prepared through the addition of discarded automotive oil and employing a microwave desulfurization process. The results showed improvements in the service performance and long-term stability of the high-dissolution rubber asphalt [32]. Yan et al. activated waste rubber using aromatic oil as an activator and performed molecular simulations. The results suggested that strong electrostatic forces existed between the rubber and the asphalt, and molecular interactions between the rubber and aromatic oil or aromatics facilitated the dissolution of rubber molecules and aromatic oil in the asphalt. Aromatic oil reduced the viscosity of the modified asphalt [33]. Chokanandsombat et al. examined the swelling and dissolution procedure of rubber in aromatic oil and its interaction mechanism. The results indicated that the swelling rate and mass loss of rubber in the oil were much higher than in asphalt and increased with the processing temperature. The dissolution of shredded rubber in oil was attributed to desulfurization, whereas its dissolution in asphalt was mainly due to depolymerization [34]. Zhao et al. pretreated waste rubber powder using aromatic oil and alkyl oil to produce RPMA and conducted macrolevel asphalt tests. The findings indicated that both aromatic oil and naphthenic oil notably reduced the viscosity of RPMA. Furthermore, pretreatment with lightweight oil improved low-temperature crack resistance and long-term stability, although the effect on high-temperature performance varied between the pre-swelling processes involving aromatic oil and naphthenic oil [35]. Zhang et al. prepared modified asphalt (ASMA) by incorporating tall oil and rubber particles with recycled aged SBS. Through the analysis of surface characteristics, morphology, and thermal behavior, the findings indicated that tall oil markedly enhanced the performance of ASMA at lower temperatures, but it negatively affected its high-temperature resistance. The secondary modification effect of recycled pitch (CR) on ASMA improved rutting resistance at high temperatures but reduced crack resistance at low temperatures and storage durability. The overall modification effect was influenced by the size and content of the rubber particles [36]. Aljarmouzi et al. added waste tire rubber along with discarded cooking oil and waste engine oil to asphalt, significantly improving the characteristics of the asphalt binder [37]. Xue et al. prepared a composite-modified asphalt using 20% rubber powder and various bio-oil contents (5%, 10%, and 15%) at low temperatures (160 °C) and for short times (30 min). Rheological and microscopic tests revealed that the recommended composition was 20% rubber and 10% renewable oil, based on the results for PG grade, elastic recovery ratio, and flow performance [38].

To conclude, modified asphalts with high rubber contents encounter significant challenges, including increased viscosity and poor workability, which severely limit their widespread application and development [39,40]. The use of lightweight oil as a regulator can effectively mitigate the high-viscosity characteristics of rubber-modified asphalt while simultaneously imparting superior comprehensive performance. This study explores the effects of various types and dosages of lightweight oil on the rheological behavior of RPMA, presents the microscopic morphology of different lightweight oil–rubber-modified asphalts, and reveals the modification mechanisms.

## 2. Objective

Traditional rubber-modified asphalt typically exhibits high viscosity and poor workability during construction. The mixing and construction temperatures of rubber-modified asphalt mixtures are excessively high. To address this limitation, this study incorporated various light oils into rubber-modified asphalt, effectively reducing its viscosity and enhancing its workability. Four types of lightweight oil were selected for this study: paraffin oil, aromatic oil, fatty acids, and tall oil. By using different types and dosages of lightweight oil–rubber composite-modified asphalts, a series of tests were performed to assess the rheological and microscopic properties. This study evaluated the effectiveness of the rubber–lightweight oil-modified asphalts; in addition, the best type of lightweight oil was selected based on the comparative analysis.

## 3. Methods

### 3.1. Materials

Neat asphalt was obtained from a production facility in Dongguan (Guangzhou) and was classified as Grade 70 petroleum asphalt. The asphalt performance was tested according to the AASHTO T specifications. The detailed properties are provided in Table 1.

The rubber powder used in this study was sourced from the Guangxi Jiaoke New Materials Technology Co., Ltd. (Nanning, China). The rubber powder was derived from truck-tire waste, with a particle size of 30 mesh (0.03 mm). The essential features of the rubber powder are provided in Table 2; all parameters met the required standards.

In this study, four types of lightweight oils were used, sourced from Nantong Yuhao Chemical Technology Co., Ltd. (Nantong, China): waste vegetable oil, paraffin oil, aromatic oil, fatty acids, and tall oil. Detailed parameters for these five types of lightweight oils are shown in Table 3, Table 4, Table 5 and Table 6.

### 3.2. Experimental Methods

#### 3.2.1. Preparation Process

The rubber content in the lightweight oil–rubber composite-modified asphalt (LRMA) was selected based on the relevant literature, with 20% crumb rubber added to the asphalt by mass [8]. Based on a review of several studies and preliminary experiments, the dosages of lightweight oil were determined to be 5% and 10% [15,23].

The preparation process for the composite-modified asphalt is outlined below. First, a predetermined quantity of neat asphalt and lightweight oil are positioned in an oven at 135 °C for 2 h to heat the neat asphalt to a free-flowing state and to remove any moisture from the lightweight oil. Then, a specific type of lightweight oil is slowly added to the neat asphalt according to the designed dosage, while stirring with a glass rod to ensure uniform mixing. The mixture is then sheared using a high-speed shear device at 135 °C and a shear rate of 3500 rpm for 5 min to obtain the lightweight oil-modified asphalt. Finally, the modified asphalt is heated to 160 °C. To prevent the formation of clumps, the crumb rubber is added in several portions to the bio-oil-modified asphalt and mixed evenly with a stirring rod. The mixture is then sheared for 30 min at a shear rate of 3500 rpm at 160 °C, resulting in the preparation of a lightweight oil–rubber composite-modified asphalt through a low-temperature, short-time shear process. Detailed information about the different types of lightweight oil–rubber-modified asphalt used in this work is presented in Table 7.

#### 3.2.2. Pressure Aging Vessel Test (PAV)

Following the guidelines of AASHTO T 240 [46], the neat asphalt and different lightweight oil–rubber composite-modified asphalts were placed in a rotating thin-film oven at 163 °C ± 5 °C for 85 min for short-term aging (rotational thin-film aging). The asphalt samples, following this short-term aging process, were then collected.

#### 3.2.3. Penetration Test

The penetration was performed according to AASHTO T 49. Penetration values at 25 °C were measured for the neat asphalt, rubber-modified asphalt, and five types of lightweight oil–rubber composite-modified asphalts. Three parallel tests were conducted for each specimen, and the mean value was taken.

#### 3.2.4. Brinell Rotary Viscosity Test (RV)

Following AASHTO T 316, Brookfield viscometers were used to measure the Brinell viscosity of different types of lightweight oil–rubber-modified asphalts. A No. 27 rotor was selected, with the rotational speed set to 20 rpm. All asphalt samples were tested at 160 °C, and each sample underwent two parallel tests to ensure data reliability.

#### 3.2.5. Bending Beam Rheological Test (BBR)

Following the guidelines of AASHTO T 313 [47], the CANNON TE-BBR test apparatus was used to assess the bending modulus of elasticity (s) and creep ratio (m) of the asphalt at a low thermal level. The temperature range for the BBR tests was −12 °C to −30 °C, with intervals of 6 °C between each test. Three independent parallel tests were performed for each temperature point to ensure the reliability and consistency of the results.

#### 3.2.6. Dynamic Shear Rheometer Test (DSR)

AASHTO T 315, for a dynamic shear rheometer, was utilized to determine the rutting index (G*/sinδ) under defined temperature and loading frequency conditions. High-temperature scanning experiments were performed on the neat asphalt and lightweight oil–rubber composite-modified asphalts. The temperature range for testing was between 46 °C and 82 °C, with scans taken every 6 °C. Each asphalt binder underwent three repeated tests to ensure the consistency and accuracy of the results.

#### 3.2.7. Multiple Stress Creep Recovery Test (MSCR)

In this study, the MSCR trial was executed in compliance with AASHTO TP 70 [48] using a DSR. The elastic and viscoelastic responses of asphalt after RTFO aging were tested. The temperature range was set between 52 °C and 76 °C, with increments of 6 °C. This test evaluated the elastic recovery and residual stress effects of the samples.

#### 3.2.8. Fourier Transform Infrared Test (FTIR)

FTIR was employed to explore the functional groups in the asphalt binders. The absorbance-versus-wavenumber curve was obtained after scanning. The mid-infrared spectrum was measured within the wavenumber spectrum of 650–4000 cm^−1^, and each specimen was scanned 32 times. The Smart iTR™ attenuated total reflection (ATR) accessory (Thermo Fisher Scientific, Waltham, MA, USA) was adopted to increase the test-result accuracy.

#### 3.2.9. Fluorescence Microscope Test (FM)

Fluorescence microscopy was utilized to examine the dispersion of rubber particles within the asphalt. A Leica LEICADM4M fluorescence microscope (Leica Microsystems, Wetzlar, Germany) with a magnification of 200× was utilized to explore the microstructure of the bio-oil–rubber composite-modified asphalts.

## 4. Results and Discussion

### 4.1. Penetration Test

**Figure 1:**
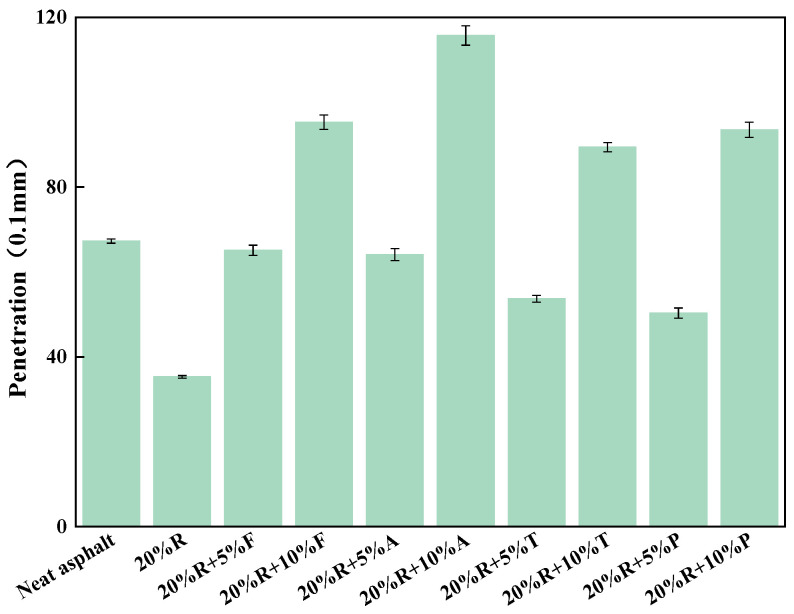
Penetration results.

The outcomes of the penetration test for various types and contents of LRMA are presented in Figure 1. As depicted in the figure, the addition of 20% crumb rubber to neat asphalt significantly reduced its penetration by 47.5%. These results indicate that the hardness of rubberized asphalt increases substantially compared to neat asphalt.

The penetration of LRMA prepared using the low-temperature, short-term shear process was significantly improved compared to 20%R. When the light oil content was 5%, the recovery ability of fatty acids and aromatic oil with respect to the penetration of rubber-modified asphalt was comparable, each increasing the penetration of 20% rubber asphalt by approximately 80%, bringing it back to the level of neat asphalt. The recovery impact of tall oil and paraffin oil on 20% rubberized asphalt was slightly lower than that of fatty acids and aromatic oils. When the light oil content was increased to 10%, the penetration of all four types of LRMA improved significantly. Among them, the penetration of 20%R + 10%A was the highest at 115, while the penetration of 20%R + 10%T was the lowest at 89.4. The penetration of 20%R + 10%F and 20%R + 10%P was approximately 95.

Upon analysis, it can be concluded that, as the light oil content increased from 5% to 10%, the seepage of fatty acid–rubber-modified asphalt increased by 46.3%, the smallest increase, while the seepage of paraffin oil–rubber-modified asphalt increased by 85.9%, the largest increase. From the penetration test results, it is evident that all light oils can significantly mitigate the increase in asphalt hardness caused by rubber. However, excessive amounts of aromatic oil can cause the penetration of aromatic oil–rubber-modified asphalt to exceed the specified limits.

### 4.2. RV Test

**Figure 2:**
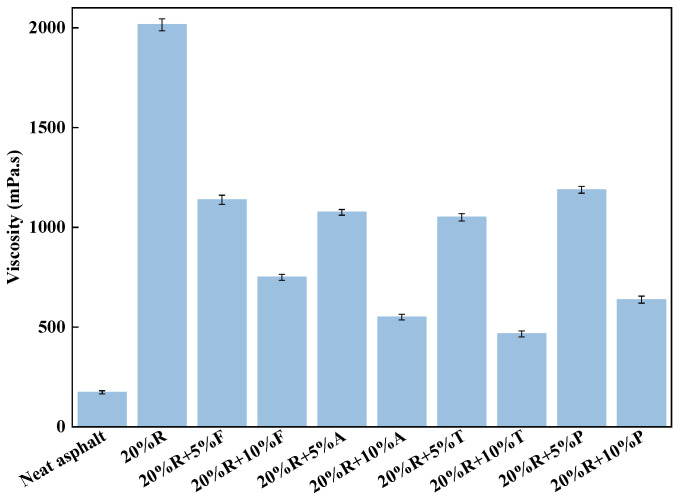
Viscosities at 160 °C for various asphalt binder types.

The Brookfield viscosity test results for various types and concentrations of LRMA at 160 °C are presented in Figure 2. The low-temperature, short-duration shear preparation process significantly reduced the viscosity. The viscosity of traditional wet rubberized asphalt with a 20% rubber content was greater than 3000 mPa·s at 160 °C, whereas the viscosity of the 20% rubber asphalt prepared using the low-temperature, short-duration shear was 2015 mPa·s, representing a 32.8% reduction.

As depicted in Figure 2, incorporating 20% crumb rubber into neat asphalt significantly increased its viscosity, with the viscosity of 20% rubber asphalt being 11.6 times greater than that of neat asphalt. However, light oils effectively mitigated the increase in viscosity caused by the addition of rubber. When the light oil content was 5%, all four types of light oils reduced the viscosity of 20% rubber asphalt by more than 40%. Among them, tall oil exhibited the greatest viscosity reduction, lowering the viscosity of rubber asphalt by 47.9%, while paraffin oil had the least effect, reducing the viscosity by 41%. When the light oil content increased to 10%, the viscosity reduction rate for fatty acid-modified asphalt decreased. The flow behavior of 20%R + 10%F was 750 mPa·s, a 62.8% reduction compared to 20% rubber asphalt, representing the smallest reduction. In contrast, the viscosity of 20%R + 10%T was 466 mPa·s at 160 °C, a 76.9% reduction compared to 20% rubber asphalt.

Additionally, when comparing the viscosity error ranges of different asphalt samples, it is evident from Figure 2 that the viscosity error of neat asphalt was the smallest, while the error range of 20% rubberized asphalt was significantly higher. This discrepancy is due to the absence of impurities in neat asphalt, resulting in minimal variation across repeated viscosity tests. In contrast, the crumb rubber added during the low-temperature, short-term preparation process does not fully dissolve, and the undissolved rubber is unevenly dispersed in the asphalt, leading to a higher viscosity error for 20% rubber asphalt. The viscosity error range for LRMA samples was notably smaller compared to that for the 20% rubber asphalt, and as the light oil content increased, the error range of LRMA viscosity gradually decreased. These results indicate that light oil promotes the dissolution and uniform diffusion of crumb rubber in asphalt. The viscosity error range for 10% tall oil was the smallest among all LRMA samples, suggesting that tall oil has the most pronounced effect on the dissolution and dispersion of crumb rubber in LRMA.

### 4.3. Rheological Tests

#### 4.3.1. Temperature Sweep

**Figure 3:**
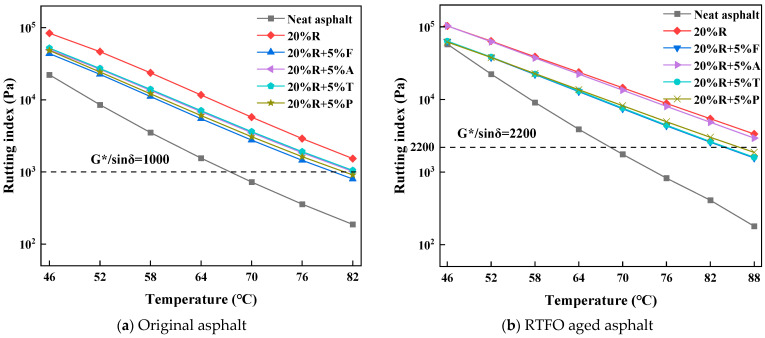
Temperature sweep of LRMA (5% light oil content).

The G*/sinδ values of LRMA with a 5% light oil content are displayed in Figure 3. As represented in the data, the G*/sinδ value of asphalt exhibited a substantial increase with the inclusion of 20% crumb rubber in neat asphalt, with the G*/sinδ of rubberized asphalt being 7.5 times greater than that of neat asphalt at 64 °C. The high-temperature performance of 20% rubberized asphalt was significantly improved compared to neat asphalt, indicating that crumb rubber enhances the high-temperature behavior of bitumen binder.

As illustrated in Figure 3a, when the light oil content was 5%, the G*/sinδ of LRMA decreased to varying extents compared to 20% rubber asphalt, but it remained much greater than that of neat asphalt, suggesting that light oil negatively impacts stability under high thermal conditions. The order of high-temperature performance for the different modified asphalts, from highest to lowest, was 20%R > 20%R + 5%T > 20%R + 5%A > 20%R + 5%P > 20%R + 5%F. The decrease in G*/sinδ with increasing scanning temperature differed among the samples, indicating that each asphalt sample had a distinct temperature sensitivity. Neat asphalt exhibited the fastest decline in G*/sinδ, indicating higher high-temperature sensitivity. The incline of the G*/sinδ curve for 20% rubber asphalt was similar to that of LRMA. This result suggests that although light oils reduce the thermal stability of asphalt binder, they do not substantially affect its temperature sensitivity.

The temperature scanning results for the asphalt samples after short-term aging are exhibited in Figure 3b. Notably, the G*/sinδ of 20%R + 5%T exhibited a significant increase after the short-term aging process, and the G*/sinδ of 20%R + 5%A increased to the same level as that of 20% rubber asphalt. According to references, aromatic oil has strong volatility. Aromatic oil and rubber composite-modified asphalt would lose a large amount of aromatic oil during 85 min of aging at 163 °C, leaving only a small amount of aromatic oil in the asphalt, which would play a role after short-duration aging.

The G*/sinδ of LRMA after aging was relatively low, but the G*/sinδ of 20% rubber asphalt and neat asphalt increased significantly. Meanwhile, the steepness of the G*/sinδ curve for neat asphalt after short-term aging was markedly steeper contrasted with that of non-aged asphalt, whereas the slope for LRMA showed minimal change. This indicates that the sensitivity of neat asphalt to temperature was notably heightened following short-term aging. These results suggest that light oil significantly enhances the aging resistance of asphalt.

**Figure 4:**
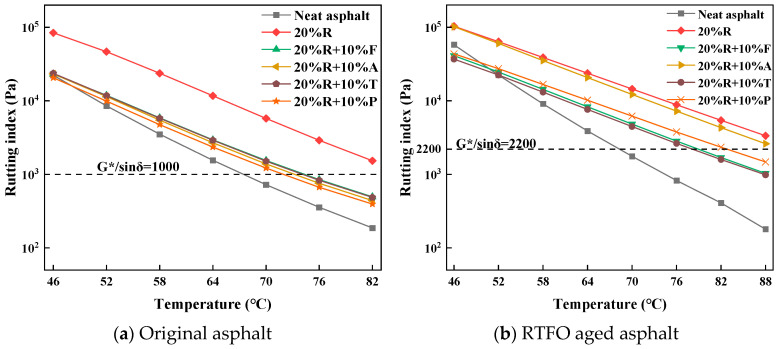
Temperature sweep of LRMA (10% light oil content).

The G*/sinδ values for the bitumen samples are presented in Figure 4. A comparison between Figure 3a and Figure 4a reveals that the G*/sinδ values for the four types of LRMA with a 10% light oil content were significantly reduced compared to those with a 5% light oil content. The capability under elevated temperatures of the four types of LRMA with a 10% light oil content is ranked as follows: 20%R > 20%R + 10%T > 20%R + 10%F > 20%R + 10%A > 20%R + 10%P. Among the LRMA with a 5% light oil content, fatty acid–rubber-modified asphalt exhibited the poorest high-temperature performance; however, when the light oil content was increased to 10%, its high-temperature stability ranked second. The decrease in high-temperature characteristics for fatty acid–rubber-modified asphalt was lower than that for aromatic and paraffin oil composite-modified asphalts with increased dosages. Additionally, no significant change in the G*/sinδ slope was observed between the four types of modified asphalts with a 10% light oil content compared to those with a 5% light oil content. These results suggest that the light oil content has a minimal effect upon the thermal sensitivity of bitumen.

The G*/sinδ results for the light oil–rubber advanced asphalt composite with a 10% light oil content after short-term aging are provided in Figure 4b. The G*/sinδ values for 20%R + 10%A were consistent with those for 20%R + 5%A, both showing a significant increase after short-term aging. The G*/sinδ values of 20%R + 5%A and 20%R + 10%A were slightly lower than those observed for the 20% rubber-modified asphalt. These findings support the previous analysis: aromatic oil in aromatic oil–rubber composite-modified asphalt volatilizes in large quantities during short-term aging, leaving only a small amount of aromatic oil to contribute to the modified asphalt after aging.

By comparing the G*/sinδ values of the asphalt samples under short-process aging with those of the original samples, it is evident that the G*/sinδ of LRMA with a 10% light oil content did not show a significant increase. Among them, the G*/sinδ of 20%R + 10%T after short-duration aging exhibited the smallest increase, indicating that toluene oil plays an essential function in augmenting the anti-aging properties of asphalt. At 46 °C, the G*/sinδ of neat asphalt was elevated compared to that of LRMA with a 10% light oil content. However, as the temperature rose to 58 °C, the G*/sinδ of neat asphalt decreased below that of all the LRMA samples, indicating that neat asphalt exhibited higher temperature sensitivity than all the LRMA samples after short-duration aging. The temperature sensitivity of the LRMA remained at a good level even after aging.

From the preceding analysis, the evidence suggests that the high-temperature behavior and aging resistance of 20%R + 5%T and 20%R + 10%T are superior to those of other types of LRMA. Therefore, toluene oil exerts a minimal influence on the high thermal properties, while enhancing its aging resistance. Table 8 presents the PG grades for the various kinds of asphalt samples.

#### 4.3.2. MSCR Test

**Figure 5:**
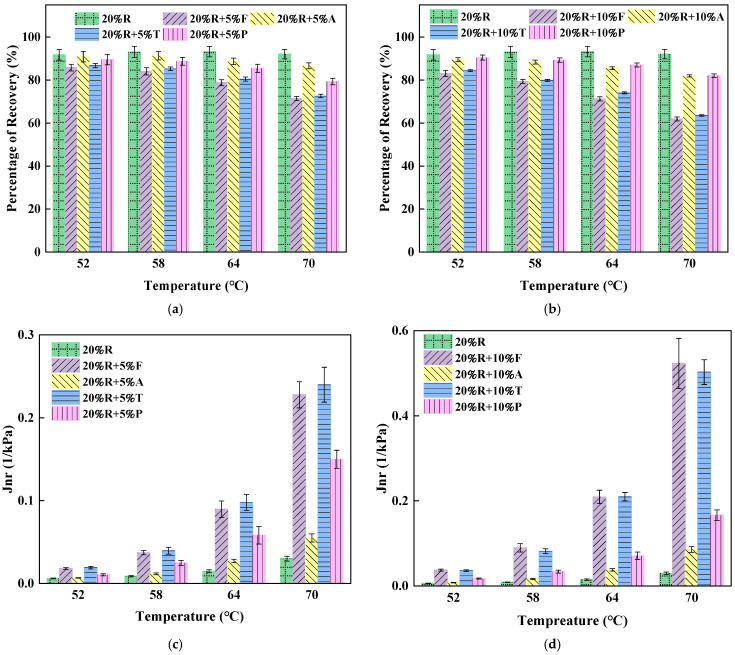
MSCR test results of difference light oil–crumb rubber composite-modified asphalts at 0.1 kPa stress level. (**a**) Effect of 5% lightweight oil dosage on asphalt elastic recovery rate. (**b**) Effect of 10% lightweight oil dosage on asphalt elastic recovery rate. (**c**) Effect of 5% lightweight oil dosage on asphalt non-recoverable creep strain. (**d**) Effect of 10% lightweight oil dosage on asphalt non-recoverable creep strain.

The percentage of recovery values (R) and Jnr results for each asphalt sample under 0.1 kPa stress are presented in Figure 5. Since the MSCR results for neat asphalt differ significantly from those for modified asphalts, they are presented separately in Table 9.

From Figure 5a,b, it can be observed that at 64 °C, the elastic recovery rate of 20%R was 35 times that of the neat asphalt. The elastic recovery rate of LRMA (lightweight oil–rubber-modified asphalt) decreased to varying extents compared to 20%R, suggesting that lightweight oil negatively affects the elastic properties of asphalt. At a test temperature of 70 °C, the elasticity behavior of neat asphalt dropped to 0%. The elastic recovery rate of 20%R remained relatively stable across all test temperatures, showing no significant decline at higher test temperatures, and remained at 90% at 70 °C. The elastic recovery rate of LRMA with different types and dosages of lightweight oil showed the same trend with increasing test temperatures. Under low-temperature conditions from 52 °C to 64 °C, the elastic recovery rate of 20%R + 10%P decreased from 90.46% to 87.04%, remaining relatively stable. However, under the higher-temperature conditions from 64 °C to 70 °C, the elastic recovery rate of 20%R + 10%P dropped from 87.04% to 82.05%, showing a significant decrease.

Simultaneously, by comparing the elastic recovery rates of LRMA with varying dosages of the same type in Figure 5, it can be seen that at 64 °C the elastic recovery rate of 20%R + 5%T was 80.48%, while that of 20%R + 10%T was 74.04%, a decrease of 6.44%. These findings suggest that at temperatures below 64 °C, increasing the dosage of lightweight oil does not lead to a substantial decline in the elastic recovery rate of asphalt. However, under the high-temperature conditions at 70 °C, the elastic recovery rate of 20%R + 5%T was 72.62%, while that of 20%R + 10%T was 63.53%, a decrease of 9.09%. At higher temperatures, lightweight oil significantly reduces the elastic recovery rate of lightweight oil–crumb rubber composite-modified asphalt, although the elastic recovery rate of the 10% lightweight oil content LRMA remained at a relatively high level of over 60% at 70 °C. Comparing the elastic recovery rates across asphalt samples at varying temperatures revealed that lightweight oil exerts a less pronounced impact on the elastic behavior of asphalt at lower temperatures but a stronger adverse effect at higher temperatures.

Under a stress of 0.1 kPa, the order of elastic recovery rates from largest to smallest was as follows: 20%R > 20%R + 5%A > 20%R + 5%P > 20%R + 10%A > 20%R + 10%P > 20%R + 5%T > 20%R + 5%F > 20%R + 10%T > 20%R + 10%F. At 64 °C, the elastic recovery rate of 20%R + 5%A was 88.72% and that of 20%R + 10%A was 85.55%, which is close to the 91.75% elastic recovery rate of 20%R. This is because of the volatilization of aromatic oil during short-term aging. At 64 °C, the elastic recovery rate of 20%R was 87.04%, remaining at a high level, indicating that paraffin oil can effectively maintain the elastic properties of asphalt.

The Jnr values for various asphalt samples at a 0.1 kPa stress level are displayed in Figure 5c,d. As the temperature rises between 52 °C and 70 °C, the Jnr value of neat asphalt increases from 0.3232 to 5.0773, showing the largest increase. At 70 °C, the Jnr value of 20%R is 0.0029, much inferior to that of neat asphalt and almost negligible. The Jnr values of 20%R + 5%A and 20%R + 5%P increase slightly with temperature, the Jnr values at 70 °C being 0.0549 and 0.1523, respectively, both lower than the Jnr value of neat asphalt at 52 °C. These findings suggest that crumb rubber-modified asphalt improves the asphalt’s resistance to permanent deformation, and 20%R + 5%A and 20%R + 5%P show better resistance to permanent deformation at 70 °C than neat asphalt at 52 °C. This is consistent with the conclusions drawn from Figure 5a,b.

**Figure 6:**
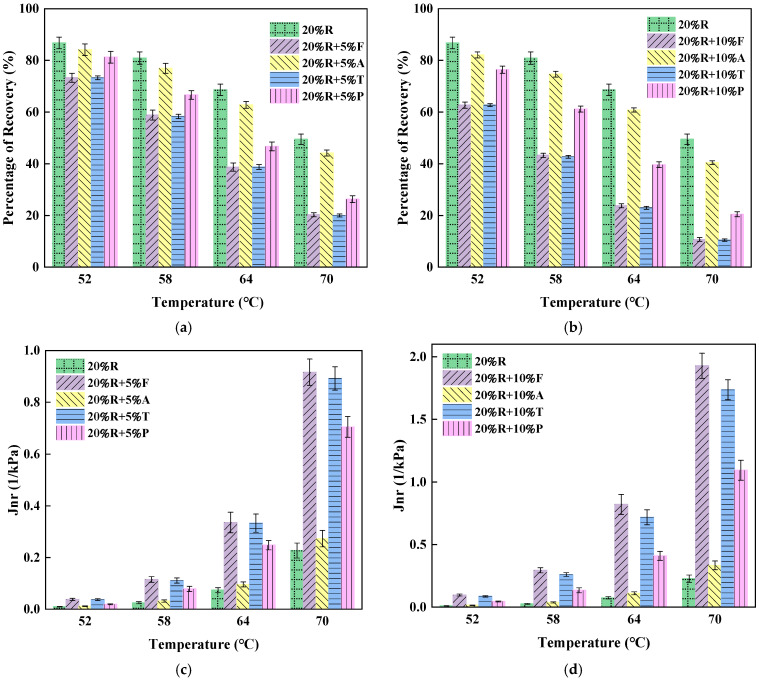
MSCR results of different light oil–crumb rubber composite-modified asphalts at 3.2 kPa stress level. (**a**) Effect of 5% lightweight oil dosage on asphalt elastic recovery rate. (**b**) Effect of 10% lightweight oil dosage on asphalt elastic recovery rate. (**c**) Effect of 5% lightweight oil dosage on asphalt non-recoverable creep strain. (**d**) Effect of 10% lightweight oil dosage on asphalt non-recoverable creep strain.

Figure 6 illustrates the elastic recovery percentage and Jnr values for asphalt samples under 3.2 kPa conditions. The MSCR results for neat asphalt at the 3.2 kPa stress level are provided in Table 10. A comparison of Figure 5 and Figure 6 shows that the elastic recovery percentage of asphalt samples at high stress levels decreases significantly compared to those at lower stress levels, with a more pronounced decrease as the temperature increases.

At the 3.2 kPa stress level, with an increase in thermal levels between 52 °C and 70 °C, while the elastic recovery of 20%R decreases from 86.75% to 49.52%. These findings suggest that, with conditions of elevated temperature and high stress, the elastic properties of bitumen degrade substantially; however, modification with crumb rubber contributes to a significant enhancement in the asphalt’s elastic characteristics.

The elastic recovery rates of the asphalt samples at the 3.2 kPa stress level, listed in descending order, were as follows: 20%R > 20%R + 5%A > 20%R + 10%A > 20%R + 5%P > 20%R + 10%P > 20%R + 5%T > 20%R + 5%F > 20%R + 10%T > 20%R + 10%F. At 64 °C, the elastic recovery rate for 20%R + 5%P was 46.71%, and for 20%R + 10%P it was 39.62%. Paraffin oil–crumb rubber composite-modified asphalt demonstrates good retention of elastic properties even under high-stress conditions.

The Jnr values of different asphalt samples at the 3.2 kPa stress level are shown in Figure 6c,d. At the temperature of 58 °C, the Jnr values of LRMA are very low, with 20%R + 10%P exhibiting the highest Jnr value of 0.2939, which is only 31% of the Jnr value of neat asphalt. Nevertheless, as the temperature increases to 70 °C and the light oil content increases to 10%, the Jnr values of LRMA increase significantly. The Jnr value of 20%R + 10%F increases by 110.4% compared to 20%R + 5%F, marking the greatest increase. The Jnr value of 20%R + 10%P increases by 55.3% compared to 20%R + 5%P, the smallest increase. These results indicate that, at high stress levels, the increase in light oil content significantly reduces the asphalt’s capacity to resist permanent deformation.

The MSCR test can be used to classify different asphalt samples according to traffic grade, with the specific grading results shown in Table 11. From the traffic grade classification results, it can be observed that 20%R, 20%R + 5%A, and 20%R + 10%A exhibit the strongest load-bearing capacity, with a grade of “Extremely Crowded (E)” at 70 °C. The 20%R + 5%P and 20%R + 10%P asphalts also have a grade of “Extremely Crowded (E)” at 64 °C, indicating that paraffin oil helps maintain better elastic properties in the modified asphalt.

#### 4.3.3. BBR Test

**Figure 7:**
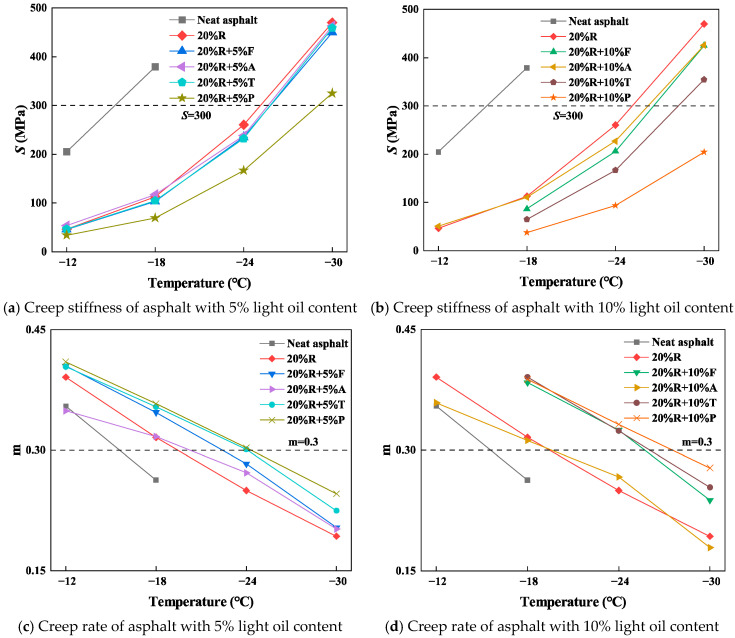
BBR test results of different light oil–crumb rubber composite-modified asphalts.

The BBR test results of the bitumen specimens in low-temperature environments are shown in Figure 7. Neat asphalt did not comply with the specification requirements at −18 °C. At −12 °C, 20%R + 10%F, 20%R + 10%T, and 20%R + 10%P all experienced significant deformation during the BBR test, making it impossible to obtain test data.

As presented in Figure 7a,b, the creep stiffness of 20%R significantly decreases compared to neat asphalt. At −12 °C, the creep stiffness of 20%R is 46.4 MPa, a 77.6% decrease compared to neat asphalt, suggesting that rubber powder can substantially upgrade the asphalt’s low-temperature anti-cracking property. The creep stiffness of LRMA decreases further, with paraffin oil having the greatest impact on reducing asphalt creep stiffness. The creep stiffness of 20%R + 5%P is 34.3 MPa, a 20% reduction compared to 20%R. The combined influence of light oil and crumb rubber can substantially elevate the cracking resistance of bitumen in low-temperature environments. The creep stiffness values for 20%R + 5%F, 20%R + 5%A, and 20%R + 5%T are 45.7 MPa, 53.5 MPa, and 46.3 MPa, respectively, which are close to that of 20%R.

As the BBR test temperature decreases to −18 °C, the creep stiffness of neat asphalt is 379.4 MPa, an increase of 84.9%. The increase in creep stiffness for 20%R and LRMA is considerably below that of neat asphalt. Both crumb rubber and light oil lead to a reduction in the temperature sensitivity of asphalt in cold environments. The creep stiffness of 20%R + 5%P is 69.7 MPa, the lowest rate of increase, indicating that paraffin oil provides excellent low-temperature performance for asphalt. For the 10% light oil–crumb rubber asphalt, the increase in creep stiffness is even lower compared to the 5% light oil–crumb rubber asphalt. The creep stiffness ranking for the asphalt samples is presented below: 20%R + 10%P < 20%R + 10%T < 20%R + 10%F < 20%R + 10%A < 20%R. When the test temperature reaches −30 °C, only 20%R + 10%P meets the specification requirements with an S-value of 204 MPa, which is below the 300 MPa limit. Asphalt with a high paraffin oil–crumb rubber content exhibits the strongest low-temperature cracking resistance.

As shown in Figure 7c,d, at the test temperature of −12 °C, the m-values for 20%R + 5%A and 20%R + 10%A are analogous to that of neat asphalt, while both are lower than that of 20%R. As the test temperature decreases, the rate of decrease in m-values for 20%R + 5%A and 20%R + 10%A is slower than for 20%R, suggesting that aromatic oil reduces the stress dissipation capacity of bitumen in cool environments while also reducing its temperature sensitivity. At a test temperature of −18 °C, the m-values for neat asphalt are 0.263, which does not meet the required standards. However, the m-values for 20%R and LRMA are both above 0.3. The test outcomes suggest that the combined effect of crumb rubber and light oil can significantly improve the stress dissipation capacity of neat asphalt in cold conditions. As the light oil content rises, the m-values of LRMA increase, suggesting that a higher light oil content significantly enhances the asphalt’s ability to dissipate low-temperature stress. At a test temperature of −24 °C, the m-values for 20%R + 5%T, 20%R + 10%T, 20%R + 5%P, 20%R + 10%P, and 20%R + 10%F are 0.301, 0.324, 0.303, 0.332, and 0.325, respectively, all meeting the specification requirement of m ≥ 0.3. However, when the temperature is −30 °C, none of the asphalt samples meet the specification requirements.

PG grading of the asphalt samples was conducted, and the PG grading results for all the asphalt samples are provided in Table 12. The PG grading outcomes reveal that the paraffin oil–crumb rubber composite-modified asphalt exhibits excellent low-temperature performance, with 20%R + 5%T showing the greatest advancement in performance.

### 4.4. FTIR Test

**Figure 8:**
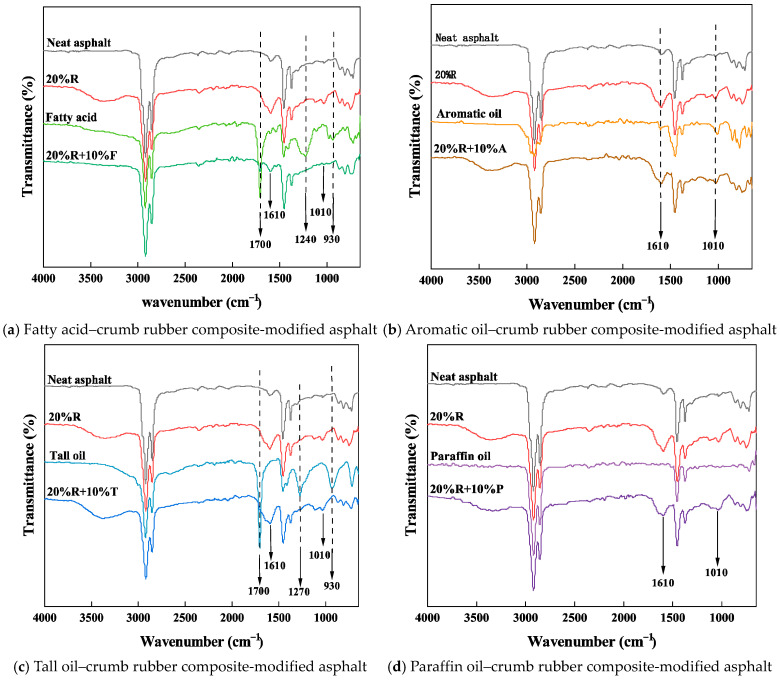
Infrared spectra of various formulations of light oil–crumb rubber composite-modified asphalt.

This research analyzed the physical and chemical alterations in asphalt during the modification process using infrared spectroscopy of neat asphalt, 20%R, four types of light oils, and LRMA. The infrared spectra of each sample are shown in Figure 8. A comparison revealed that no new absorption peaks emerged in the spectra of LRMA, 20%R, and the light oils used for modification. This suggests that, during the modification process, the light oil and crumb rubber in the asphalt form a physical blend without undergoing chemical reactions.

As can be seen in Figure 8, four types of composite-modified asphalts and crumb rubber-modified asphalts exhibited strong new absorption peaks at 1610 cm^−1^ and 1010 cm^−1^ compared to neat asphalt. The peak at 1610 cm^−1^ is attributed to the C=C tensile vibration in toluene, and the peak at 1010 cm^−1^ is due to the S=O group vibration, which is caused by the crumb rubber.

Compared to neat asphalt, the infrared spectrum of fatty acids exhibits distinct adsorption at 1700 cm^−1^, 1240 cm^−1^, and 930 cm^−1^. The peak at 1700 cm^−1^ is due to the C=O tensile vibration in esters of saturated fatty acids, the peak at 1240 cm^−1^ is caused by the C-C tensile vibration in ester groups, and the peak at 930 cm^−1^ is attributed to the C-H tensile vibration. The fatty acid–crumb rubber-modified asphalt displays a prominent absorption peak at 1700 cm^−1^, while the peaks at 1240 cm^−1^ and 930 cm^−1^ are absorbed by the crumb rubber and asphalt. This indicates that fatty acids undergo physical modification within the asphalt, with the light components of the fatty acids being absorbed by the rubber and bitumen.

The infrared spectrum of aromatic oil exhibits distinct adsorption peaks at 1610 cm^−1^ and 1010 cm^−1^, which are consistent with the characteristic peaks observed in crumb rubber asphalt. This is because aromatic oils, being aromatic compounds, are highly compatible with crumb rubber, which is commonly used in crumb rubber processing. Additionally, the pyrolysis of crumb rubber can yield aromatic oils. Thus, the aromatic oil in asphalt also undergoes physical modification and is incorporated into the crumb rubber during the modification process.

Tall oil primarily consists of fatty acids and rosin acid, so its infrared spectrum shows characteristic absorption peaks at 1700 cm^−1^ and 930 cm^−1^, owing to fatty acids, and at 1270 cm^−1^ as a result of the C-O tensile vibration in the -COOH group of rosin acid. Therefore, the infrared spectrum of tall oil–crumb rubber-modified bitumen is quite similar to that of fatty acid–crumb rubber-modified asphalt, and both are considered physical modifications.

The absorption peaks in paraffin oil appear at the same positions as in neat asphalt. Thus, the infrared spectrum of paraffin oil–rubber-modified asphalt, like that of rubber-modified asphalt, only displays additional adsorption peaks at 1610 cm^−1^ and 1010 cm^−1^. The infrared analysis indicates that, with the shear alteration process, the lighter parts of the light oils are incorporated into both the crumb rubber and the asphalt.

### 4.5. FM Test

**Figure 9:**
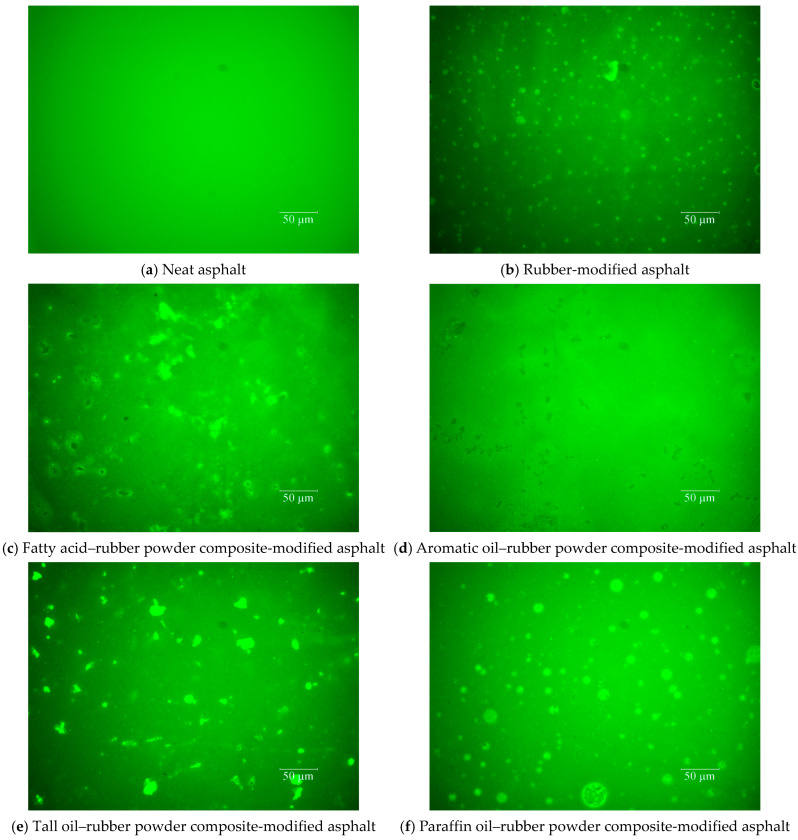
Microstructures of different light oil–rubber powder composite-modified asphalts.

The infrared spectral results indicate that the light oils and crumb rubber only undergo physical blending in the asphalt. To gain deeper insight into the microstructure of LRMA, this study used fluorescence microscopy to examine the microstructures of neat asphalt, rubber-modified asphalt, and four types of LRMA. The findings of these observations are presented in Figure 9.

As illustrated in Figure 9a, neat asphalt did not exhibit any fluorescent substances. In the microscopic structure of rubber-modified asphalt, depicted in Figure 9b, the crumb rubber is evenly distributed within the bitumen. However, the particles of rubber powder in the rubber-modified asphalt are relatively small, and the swelling reaction of the rubber powder in the modified asphalt is not very pronounced.

In contrast to the microstructure of rubber powder-modified asphalt, the swelling reaction of rubber powder in the light oil–rubber-modified asphalt is more significant. Different types of light oils enhance the expansion of crumb rubber to varying degrees.

As illustrated in Figure 9c, the high-brightness fluorescent substances in the fatty acid–crumb rubber composite-modified asphalt represent fully swollen crumb rubber, although a small fraction of the crumb rubber shows insufficient swelling, appearing black. In Figure 9d, the crumb rubber in the aromatic oil–crumb rubber composite-modified asphalt is entirely represented as large black substances, indicating that the aromatic oil has a limited effect on promoting the expansion of rubber crumbs in asphalt, and the extent of swelling of rubber powder in aromatic oil–rubber powder-modified asphalt is relatively low. In Figure 9e, the crumb rubber in the tall oil–rubber powder composite-modified asphalt is fully swollen and appears as high-brightness silver substances, with a brightness greater than that of the fluorescent substances in fatty acid–rubber powder composite-modified asphalt. In Figure 9f, the crumb rubber appears as circular fluorescent substances, but with distinct boundaries, indicating that the crumb rubber in paraffin oil–rubber powder composite-modified asphalt is not fully swollen. Adding oil could increase light components in asphalt, promote CR swelling and breaking, and reduce the intermolecular force between SARA and CR.

The swelling degrees of different light oil–rubber powder composite-modified asphalts are as follows: tall oil–rubber powder composite-modified asphalt > fatty acid–rubber powder composite-modified asphalt > paraffin oil–rubber powder composite-modified asphalt > aromatic oil–rubber powder composite-modified asphalt > rubber powder-modified asphalt. The order of the swelling degrees of the light oils in the modified asphalts is inversely related to their elastic properties, suggesting that incompletely swollen crumb rubber can provide good elastic performance in asphalt. The microstructure observed through fluorescence microscopy verifies that different light oils promote the dissolution of rubber powder in asphalt to varying degrees. The incompletely swollen rubber powder can endow the light oil–crumb rubber composite-modified asphalt with good elastic properties.

## 5. Conclusions

This study prepared composite-modified asphalts using various types and dosages of light oils and crumb rubber. Viscosity tests evaluated their workability, while DSR and BBR characterized their high- and low-temperature performance. PG grading was conducted, and the modification mechanisms were analyzed using FTIR and fluorescence microscopy.

(1) Different light oils reduced the viscosity of rubber-modified asphalt to varying degrees. At a 5% dosage, all four oils lowered the viscosity by approximately 40%. At 10%, tall oil achieved the highest reduction (76.9%), while fatty acids were least effective (62.8%).
(2) Paraffin oil best preserved the asphalt’s elasticity. The ranking of elastic performance was as follows: RMA > aromatic oil–RMA > paraffin oil–RMA > tall oil–RMA > fatty acids–RMA. Both 20%R + 5%P and 20%R + 10%P met the “E” traffic level at 64 °C.
(3) The PG grade of neat asphalt was 64-22, while 20%R reached PG 82-28, showing significant improvement. Tall oil further enhanced low-temperature cracking and aging resistance without compromising high-temperature performance, with 20%R + 5%T reaching PG 82-34.
(4) During shearing, light oil components dissolved into both asphalt and rubber. Dissolution exhibited the following order: tall oil–RMA > fatty acid–RMA > paraffin oil–RMA > aromatic oil–RMA > RMA. The swelling capacity was inversely related to elasticity, indicating that partially undissolved rubber contributed to better elastic recovery.
(5) Tall oil–RMA showed the best overall performance, promoting the highest rubber dissolution and exhibiting superior high- and low-temperature behavior. Thus, tall oil is recommended as an optimal additive for preparing light oil–crumb rubber-modified asphalt.

## 6. Future Research Work

This study investigated the macroscopic properties and microscopic mechanisms of four types of bio-oil–rubber-modified asphalts. Future research will focus on evaluating the fatigue performance, mechanical characteristics, and service behavior of corresponding asphalt mixtures, thereby providing data support and theoretical guidance for the development and practical implementation of bio-based modified asphalts.

## Figures and Tables

**Table 1:** Basic performance indicators of neat bitumen.

Test Items	Measured Value	Reference Standard
Penetration at 25 °C	68.1 (0.1 mm)	AASHTO T 49 [41]
Softening point	49.5 (°C)	AASHTO T 53 [42]
Ductility at 10 °C	32.2 (mm)	AASHTO T 51 [43]
Viscosity at 135 °C	470.9 (mPa⋅s)	AASHTO T 316 [44]
G*/sinδ	1266.3 (64 °C)	AASHTO T 315 [45]
G*/sinδ of aged asphalt	2474.2 (64 °C)	AASHTO T 315 [45]

**Table 2:** Basic physical parameters of rubber powder.

Test Items	Standards	Result
Density	≤1.2 (g/cm^3^)	1.1
Water content	≤1.0 (%)	0.6
Metal content	≤10 (%)	8
Acetone extract	≤10 (%)	6
Rubber hydrocarbon content	≥48 (%)	58
Carbon black content	≥26 (%)	34

**Table 3:** Basic parameters of aromatic oil.

Test Items	Unit	Test Result
Density (15 °C)	g/mL	1.01
Dynamic viscosity (100 C)	mm/s^2^	24.6
Aromatic hydrocarbon content	%	95
Aromaticity	%	35
Water content	%	0.03

**Table 4:** Basic parameters of tall oil.

Test Items	Unit	Test Result
Density (15 °C)	g/mL	0.96
Acid value	mg KOH/g	191.8
Rosin acid content	%	29.45
Fatty acid content	%	69.33
Saponification value	mg KOH/g	184.6

**Table 5:** Basic parameters of fatty acids.

Test Items	Unit	Test Result
Density (15 °C)	g/mL	0.94
Acid value	mg KOH/g	173
Hydroxyl value	mg KOH/g	147
Iodine value	mg KOH/g	89.1
Saponification value	mg KOH/g	185.2

**Table 6:** Basic parameters of paraffin oil.

Test Items	Unit	Test Result
Density (15 °C)	g/mL	0.85
Dynamic viscosity (100 °C)	mm/s^2^	8.12
Flash point	°C	145
Pour point	°C	−23
Water content	%	0

**Table 7:** Detailed information about the samples.

Rubber Content	Light Oil Type	Light Oil Content	Abridgement
20%	-	0%	20%R
	Fatty acids	5%	20%R + 5%F
		10%	20%R + 10%F
	Aromatic oil	5%	20%R + 5%A
		10%	20%R + 10%A
	Tall oil	5%	20%R + 5%T
		10%	20%R + 10%T
	Paraffin oil	5%	20%R + 5%P
		10%	20%R + 10%P

**Table 8:** High-temperature PG grades of different LRMA samples.

Asphalt Kind	Neat Asphalt	20%R + 5%F	20%R + 5%A	20%R + 5%T	20%R + 5%P
High-temperature PG grade	PG64	PG76	PG82	PG82	PG76
Asphalt type	20%R	20%R + 10%F	20%R + 10%A	20%R + 10%T	20%R + 10%P
High-temperature PG grade	PG82	PG70	PG70	PG70	PG70

**Table 9:** MSCR results of neat asphalt at 0.1 kPa stress level.

Temperature (°C)	R (%)	Jnr (1/kPa)
52	12.08	0.3232
58	6.52	0.8704
64	2.51	2.1841
70	0	5.0773

**Table 10:** MSCR test results of neat asphalt at 3.2 kPa stress level.

Temperature	R	Jnr
52 °C	8.73%	0.3392 (1/kPa)
58 °C	2.24%	0.9505 (1/kPa)
64 °C	0%	2.4019 (1/kPa)
70 °C	0%	5.5113 (1/kPa)

**Table 11:** Different grades of LRMA for traffic loading.

Asphalt Type	Traffic Load Grades		
	58 °C	64 °C	70 °C
Neat asphalt	V	S	F
20%R	E	E	E
20%R + 5%F	E	E	F
20%R + 10%F	E	F	F
20%R + 5%A	E	E	E
20%R + 10%A	E	E	E
20%R + 5%T	E	E	F
20%R + 10%T	E	F	F
20%R + 5%P	E	E	F
20%R + 10%P	E	E	F

Note: S, V, and E stand for standard, very high, and extremely high traffic loading, respectively. F means the asphalt exhibits failure at this temperature.

**Table 12:** PG grades of different LRMAs.

Asphalt Type	Neat Asphalt	20%R + 5%F	20%R + 5%A	20%R + 5%T	20%R + 5%P
PG grade	PG 64-22	PG 76-28	PG 82-28	PG 82-34	PG 76-34
Asphalt type	20%R	20%R + 10%F	20%R + 10%A	20%R + 10%T	20%R + 10%P
PG grade	PG 82-28	PG 70-34	PG 70-28	PG 70-34	PG 70-34

## Data Availability

The original contributions presented in this study are included in the article. Further inquiries can be directed to the corresponding author.
